# Methylation of guar gum for improving mechanical and barrier properties of biodegradable packaging films

**DOI:** 10.1038/s41598-019-50991-7

**Published:** 2019-10-10

**Authors:** Jyoti Tripathi, Rupali Ambolikar, Sumit Gupta, Dheeraj Jain, Jitendra Bahadur, Prasad Shekhar Variyar

**Affiliations:** 1 Food Technology Division, Bhabha Atomic Research Centre, Mumbai, 400085 India; 2 Chemistry Division, Bhabha Atomic Research Centre, Mumbai, 400085 India; 3 Solid State Physics Division, Bhabha Atomic Research Centre, Mumbai, 400085 India

**Keywords:** Biopolymers, Mechanical properties

## Abstract

Improving functional properties of biopolymers for use as environment friendly packaging is an area of current interest. Biodegradable films with improved barrier and mechanical properties were prepared from methylated guar gum. Methylation resulted in structural modification of guar gum (GG) promoting greater crystallization thereby enhancing thermal stability towards decomposition. Reduction in radius of gyration (R_*g*_), weight average molecular weight (*M*_*w*_), and an increase in polydispersity index (PDI) were also observed due to methylation. Methylated guar gum (MGG) films exhibited 40% lower water vapor transmission rate (WVTR) as compared to control purified guar gum (PGG) films. Films prepared by partial replacement of PGG with MGG (10, 25, 50, 75 and 100% w/w) showed gradual improvement in percent elongation. The study gives an insight on the role of methylation in enhancing barrier and mechanical properties of GG based biodegradable films for possible application in food packaging.

## Introduction

Serious environmental hazards posed by conventional petroleum-based packaging materials have led to an increased interest in recent years towards development of biodegradable packaging materials. Suitable candidates for developing biodegradable films include natural polymers such as carbohydrates, proteins and lipids. Most promising among these are carbohydrates due to their excellent film forming properties. Guar gum (GG) is a water soluble galactomannan, which is extracted from the seeds of a legume plant *Cyamopsis tetragolonoba*. Chemically, guar gum has a backbone of β-D-1,4-linked mannose residues, with side chains of α-D-galactose residues linked to O-6 of every second mannose residue^1^. Wide availability and excellent film forming properties has led to the use of this biopolymer as a promising raw material for development of biodegradable packaging films.

Lower mechanical strength and high permeability to water vapor as compared to petrochemical-based plastics are some of the drawbacks, which limit commercial use of biopolymers. Several physical methods such as UV curing, thermal processing^2^, gamma irradiation^3,4^, and addition of various additives^5^ have been demonstrated to be useful for overcoming these limitations. We had earlier demonstrated that guar gum subjected to low dose gamma radiation (500 Gy) resulted in 33% improvement in tensile strength as compared to control gaur gum based films. This improvement was noted due to ordering in polymer chains as a result of low dose gamma radiation^4^. GG nano-composites with natural refined bentonite clay (Nanofil-16) had 104% higher tensile strength and 24% lower water vapor transmission rate in comparison to control^6^. In a recent study, concentration of various additives such as glycerol, beeswax, nanoclay and tween-80 was optimized using response surface methodology to improve mechanical and barrier properties of GG films^7^. Enzymatic modification has also been used for improving mechanical properties of GG film^8^.

Highly polar nature of carbohydrate based biopolymers including GG results in films with poor mechanical strength, barrier properties and sealability, thereby restricting their use as packaging material. Chemical modification of GG using various reagents for improving its hydrophobicity has been reported^9,10^. Guar galactomannan chemically modified with benzamide resulted in formation of water resistant film^11^. Partial methylation of guar gum has also been attempted for preparation of non-ionic derivative of guar gum^10^. These derivatives have, however, not been investigated so far for their suitability for packaging applications.

Chemical modifications could possibly lead to changes in conformation of polymer in solutions and its thermal behavior. Several investigations have shown that the conformation and morphology of polymer chains affect the physical properties of the polymer^6,12^. In the present study, the effect of methylation on thermal, mechanical and barrier properties of GG films were therefore taken up for detailed investigation.

## Methods

### Chemicals

Guar gum was procured from M/s. Jai Bharat Gums and Chemicals Ltd. (Siwani, India). Apparent viscosity of guar gum as reported by manufacturer was 2500 cps (1% w/v solution) with Brookfield LVT spindle No. 4 at 20 rpm. Glycerol and NaOH was purchased from S. D. Fine chemicals Ltd. (Mumbai, India). Methyl Iodide (>99% purity) was obtained from Loba chemie laboratory reagents and fine chemicals (Mumbai, India). Ethanol was procured from Oasis India Pvt. Ltd. (Satara, India). Acetone was purchased from SRL Pvt. Ltd. (Mumbai, India). Barium hydroxide was obtained from BDH Pvt. Ltd. All reagents except guar gum were of analytical grade and solvents were distilled before use.

### Purification of guar gum

Guar gum was purified as described earlier^4^. In brief, homogenous aqueous solution (1% w/v) of guar gum was prepared using high speed homogenization (Omnimixer, Sorvall, U.S.A.) at speed 3 for three minutes. Prepared solution was then kept for 12 h at room temperature (25 ± 2 °C) with magnetic stirring for proper hydration of guar gum. Solution was then centrifuged (Eppendorf, Germany) at 9000 rpm for 30 minutes. Ethanol was then added in a proportion of 2:1 to the supernatant and left overnight to precipitate out the purified guar gum. The precipitate was filtered out and subsequently freeze-dried (ScanVac, Denmark) to obtain purified guar gum powder. Yield of purified guar gum (PGG) obtained was 62%, which was similar to that of earlier reports^4^.

### Methylation of guar gum

Methylation was done according to procedure as described earlier^10^ with slight modifications. In brief, 1% aqueous solution (250 mL) of PGG was added with 2.5% NaOH and 2 mL methyl iodide (Supplementary Information Table S1). Reaction was carried out in aqueous medium unlike Risica *et al*.^10^, who performed in hydro-alcoholic mixture to minimize the use of chemical solvents. The reaction mixture was continuously kept on stirring under inert atmosphere of nitrogen for 2.5 h at room temperature (25 ± 2 °C). After completion of reaction, the solution was neutralized (pH~7.0) using acetic acid. Acetone (1:1) was added to reaction mixture to precipitate the product. Precipitate was freeze-dried to obtain methylated guar gum (MGG).

### Fourier transform infrared spectroscopy

FT-IR spectra of the guar gum and synthesized methyl derivatives were recorded using FT-IR spectrometer (Jasco, 4100). Forty scans were averaged at a resolution of 4 cm^−1^ in the range of 4000–650 cm^−1^. All spectra were baseline corrected and normalized for peak area.

### Determination of degree of substitution (DS)

Degree of methylation was determined using ^1^H NMR experiments. 1% solution of purified guar gum and methylated guar gum were subjected separately to acid hydrolysis using 1 N H_2_SO_4_ at 90 °C for 1.5 h. After completion of hydrolysis, the solution was neutralized (pH~7.0) with barium hydroxide. BaSO_4_ precipitate was removed by centrifugation (9000 rpm) and the supernatant was freeze-dried to obtain hydrolyzed products. The hydrolyzed samples were then dissolved in D_2_O and subjected to NMR analysis using Varian 500 MHz NMR spectrometer. The ratio of non-anomeric protons to anomeric protons in methylated guar gum was found to be higher than that in the native guar gum. The degree of substitution was calculated as reported earlier^10^ using Eq. 1.

1 $$DS=\frac{(NP(MGG)-NP(PGG))/A(PGG)}{3}$$

Where, *NP(MGG)* = non anomeric protons in methylated guar gum

*NP(PGG)* = non anomeric protons in native guar gum

*A(PGG)* = anomeric protons in native guar gum

### Rheological behavior

Rheological parameters (apparent viscosity, consistency index and flow behavior index) were determined using a rotational viscometer (DV-1, Brookfield, USA) as per procedure reported earlier^13^. In brief, 1% aqueous solutions of PGG and MGG were prepared and apparent viscosity was measured using spindle no. 4 at 20 rpm after a hydration period of 5 h. All the readings were taken in triplicate.

For determination of consistency index and flow behavior index, viscosities of the solutions (5 h after preparation of solution) were measured using spindle no. 7 at 0.5, 1.0, 2.0, 2.5, 4.0, 5.0, 10, 20, 50 and 100 rpm with shear rates 0.104, 0.209, 0.418, 0.522, 0.836, 1.045, 2.090, 4.180, 10.450 and 20.9 s^−1^, respectively. Shear rates were determined as per manufacturer specifications. Data were then fitted using power-law model as given by Eq. 2.

2 $$\sigma =K{(du/dy)}^{n}$$

Where, σ is shear stress (Nm^−2^); *du/dy* is shear rate (s^−1^); K is consistency index (Ns^n^/m^2^) and n (dimensionless) is flow behavior index (FBI).

### Viscosity average molecular weight determination

Ostwald’s viscometer was employed for determining the viscosity average molecular weights of PGG and MGG at a constant temperature (25 ± 1 °C). 0.1% solutions of PGG and MGG were prepared and their specific viscosity (*η*_*sp*_) was calculated using Eq. 3.

3 $$[{\eta }_{sp}]=\frac{(t-{t}_{0})}{{t}_{0}}$$

Where, t is time taken by polymer solution to flow through viscometer;

t_0_ is time taken by pure solvent to flow through viscometer.

Intrinsic viscosity was then calculated using Eq. 4.

4 $$[\eta ]=\frac{[{\eta }_{sp}]}{c}$$

Where, c = polymer concentration

Viscosity average molecular weight (*M*_*v*_) was further calculated using the following Eq. 5^14^:

5 $$[\eta ]=K{M}_{v}^{a}$$

Where, ‘K’ and ‘a’ are the parameters dependent on polymer-solvent pair. For guar galactomannan, values used for ‘K’ and ‘a’ were 0.72 and 5.13 × 10^−4^, respectively^15^.

### Gel permeation chromatography

Gel permeation chromatography (GPC) was employed to determine the number average molecular weight (*M*_*n*_) and weight average molecular weight (*M*_*w*_) of PGG and MGG. The methodology, HPLC system, mobile phase and separation conditions used were as per our previous report^4^. The column was calibrated using pullulan standards (Fluka Analytical, St. Louis, USA) ranging from molecular weights of 6000 to 2,560,000 Da (Supplementary Information Table S2).

### Preparation of Guar gum films

Films with PGG, MGG and mixtures of PGG and MGG in different ratios (90:10, 75:25, 50:50, 25:75:: PGG: MGG) were prepared according to procedure described earlier^4^. In brief, 150 mL of 1% aqueous solution of PGG/MGG was added with 0.6 g glycerol (40% w/w GG) as plasticizer. The films were casted on 20 × 20 cm glass plates with removable boundaries of insulating tape. Plates were then dried and conditioned as per protocol reported earlier^4^.

### Physical and mechanical properties of films

Various physical and mechanical properties of the films (Puncture strength, thickness, tensile strength (TS), Young’s modulus (E) and percent elongation at break (%E)) were measured as per American Society for Testing Materials (ASTM) standard method D882-10 and the procedure as reported previously^4^. For measurement of water vapor transmission rate (WVTR), films were sealed using adhesive tapes on mouth (area 80 cm^2^) of round cups (25 mL) having 15 mL of distilled water. Sealed cups were then kept inside desiccators at 25 ± 2 °C maintained at 87% relative humidity. The amount of water lost (g) was plotted with time and WVTR (g/m^2^/day) was calculated as slope of steady state region. Color (L*, a* and b* values) of films was measured using a colorimeter (CM-3600d Konica Minolta sensing Inc., Japan). Opacity of films was determined using Hunter lab method using Eq. 6 as given below:

6 $$Opacity=({Y}_{b}/{Y}_{w})\times 100$$

where

*Y*_*b*_ = reflectance on black standard

*Y*_*w*_ = reflectance on white standard

### Characterization of thermal behavior (DSC)

Thermal characterization of both PGG and MGG was carried out by differential scanning calorimetry (DSC 823^e^, M/s. Mettler-Toledo GmBH, Switzerland). In order to minimize adsorbed moisture, PGG and MGG powders were stored in desiccators (<5% RH) for 48 h prior to DSC sample preparation. Small amount of powders was placed in aluminum sample pans (40 μl) and weighed. Sample pans were then hermitically sealed using aluminum lids which were punctured with a needle for release of gaseous species/moisture during DSC experiments. An empty aluminum pan was used as reference pan. DSC thermograms were recorded over 35–550 °C with a heating/cooling rate of 10 °C/min under inert atmosphere (HP Ar, 60 mL/min.). The calorimeter was calibrated for heat flow and temperature accuracy using multiple standard materials (Hg, In, Sn, Pb, Zn). The residue obtained after DSC runs were stored in desiccators and weighed.

### Small-angle neutron scattering (SANS)

To investigate conformation of polymer and its chain length, SANS measurements were carried out using aqueous dispersion of PGG and methylated guar gum (MGG). PGG and MGG were dissolved in D_2_O (1% w/v) to carry out SANS measurements in order to reduce the incoherent background. SANS experiments were carried out using a conventional SANS facility operating at Dhruva Reactor, India^16^. The average wavelength (λ) of the incident neutron beam was 5.2 Å with a wavelength resolution (Δλ/λ) of approximately 15%. The scattering intensity was recorded as a function of wave vector q ( = 4πsin(θ)/λ, where 2θ is scattering angle) in the range of 0.015–0.3 Å^−1^. The corrections in the measured SANS data were made for the background, the empty cell contribution and the transmission.

### Statistical analysis

Statistical analysis was performed using DSAASTAT (Ver. 1.101)^17^. Measurement data was analyzed by analysis of variance and multiple comparisons of means were done by Duncan’s multiple range test at a significance level of 0.05.

## Results and Discussion

### Characterization of methylated guar gum

Under the experimental conditions employed, the yield of methylated guar gum (MGG) obtained was found to be 94%. However, unlike the previously reported methods^10^, wherein isopropyl alcohol or tertiary butyl alcohol were employed as reaction medium, methylation was performed in aqueous medium in the present study. The degree of substitution achieved in the earlier studies ranged from 0.1 to 1.7 while the corresponding value in the present study was 0.4 as determined by 1 H NMR. A degree of substitution comparable to the already reported data in literature was thus obtained. A protocol for green synthesis of MGG replacing the use of conventional chemical solvents was thus established.

**Figure 1:**
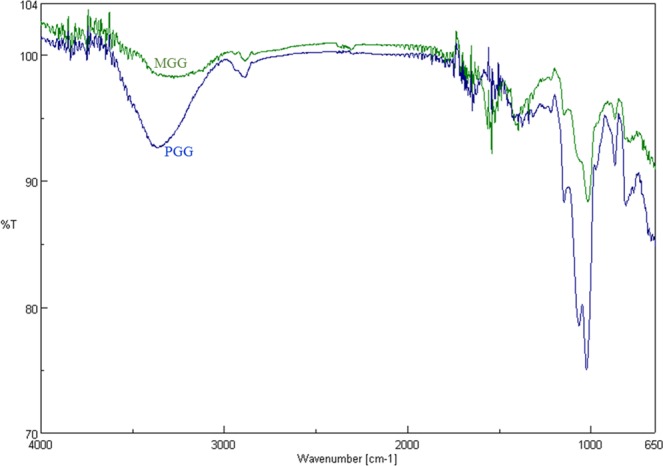
FT-IR spectra of purified guar gum (PGG) and methylated guar gum (MGG).

Fig. 1 depicts the FT-IR spectra of PGG and MGG. The hydroxyl absorption band at 3200–3600 cm^−1^ present in native guar gum shifted to higher frequencies as well as broadened in the methylated guar gum suggesting significant changes in inter- and intra-molecular hydrogen bonding and a consequent weakening in hydrogen bonds. Polysaccharides are reported to have characteristic fingerprint bands in the region 1200–1000 cm^−1^ comprising mainly of ring vibrations overlapped with stretching vibrations of (C-OH) side groups and C-O-C bonds of glycosidic bridges^18^. An absorption band at 1140 cm^−1^ corresponding to this region observed in PGG showed a reduction in intensity in MGG (Fig. 1) clearly indicating a decrease in the number of hydroxyl (-OH) groups as a result of substitution with methyl group. The intensities of bands at 1070 cm^−1^ and 1020 cm^−1^ corresponding to C6-O-C1 and alcoholic (C-O) group stretching respectively also reduced significantly.

### Effect of methylation on rheological parameters (apparent viscosity, consistency index and flow behavior index)

Significant (p < 0.05) reduction in apparent viscosity (AV), intrinsic viscosity (*η*) and viscosity average molecular weight (*M*_*v*_) was observed consequent to methylation of PGG. AV, *η* and *M*_*v*_ values for PGG were 2.350 Pa-s, 13.8 dl/g, 1.44 × 10^6^ Da while that for MGG were 0.56 Pa-s, 11.9 dl/g and 1.17 × 10^6^ Da, respectively. The observed reduction in the above parameters could possibly be accounted for either due to hydrolysis of guar gum under basic conditions employed^10^ or due to the introduction of non-polar groups consequent to methylation, resulting in reduced ability to form inter molecular hydrogen bonds. The intrinsic viscosity of guar gum (13.8 dl/g) has been earlier reported to decrease to 11.2 dl/g and 10.2 dl/g upon methylation at a degree of methylation of 0.3 and 0.6, respectively^10^. Results obtained in present work are therefore in accordance with that reported earlier.

Consistency index (CI) and flow behavior index (FBI) are two other important parameters for describing the rheological behavior of polymeric solutions. CI provides an impression of the viscosity of the solution while FBI indicates behavior under varying shear rate. A value of FBI less than 1 indicates shear thinning (pseudoplastic) while value greater than 1 shows shear thickening (dilatant) behavior of solution. Value of 1 for FBI is observed for Newtonian solutions^19^. CI of PGG was 5.58 Pa s^n^ while FBI was 0.63 indicating pseudoplastic nature of guar gum solutions. A significant (p < 0.05) effect on both CI and FBI was observed due to methylation. A reduction in CI to 1.375 Pa s^n^ with increase in FBI to 0.73 was obtained in MGG. Our results demonstrate for the first time a reduction in consistency due to methylation with solutions approaching Newtonian flow. Since CI is related to viscosity of solution, its lower value is due to observed reduction in viscosity due to methylation. In case of polymeric solutions showing pseudoplastic behavior it is postulated that under low shear, molecular chains are randomly distributed, however in high shear rates, they align themselves in direction of shear and thereby produce less resistance and consequently less apparent viscosity^20^. Generally, with lowering of molecular weight shear thinning property of solution decreases. In a previous work on irradiated guar gum^13^, it was demonstrated that radiation processing of GG reduced CI while increasing FBI as a result of reduction in AV and molecular weight. In present study, a reduction in *M*_*v*_ due to methylation was observed which has led to increase in FBI. To further analyze the effect of methylation on GG, samples were evaluated using gel permeation chromatography.

### Molecular weight determination by Gel permeation chromatography (GPC)

**Figure 2:**
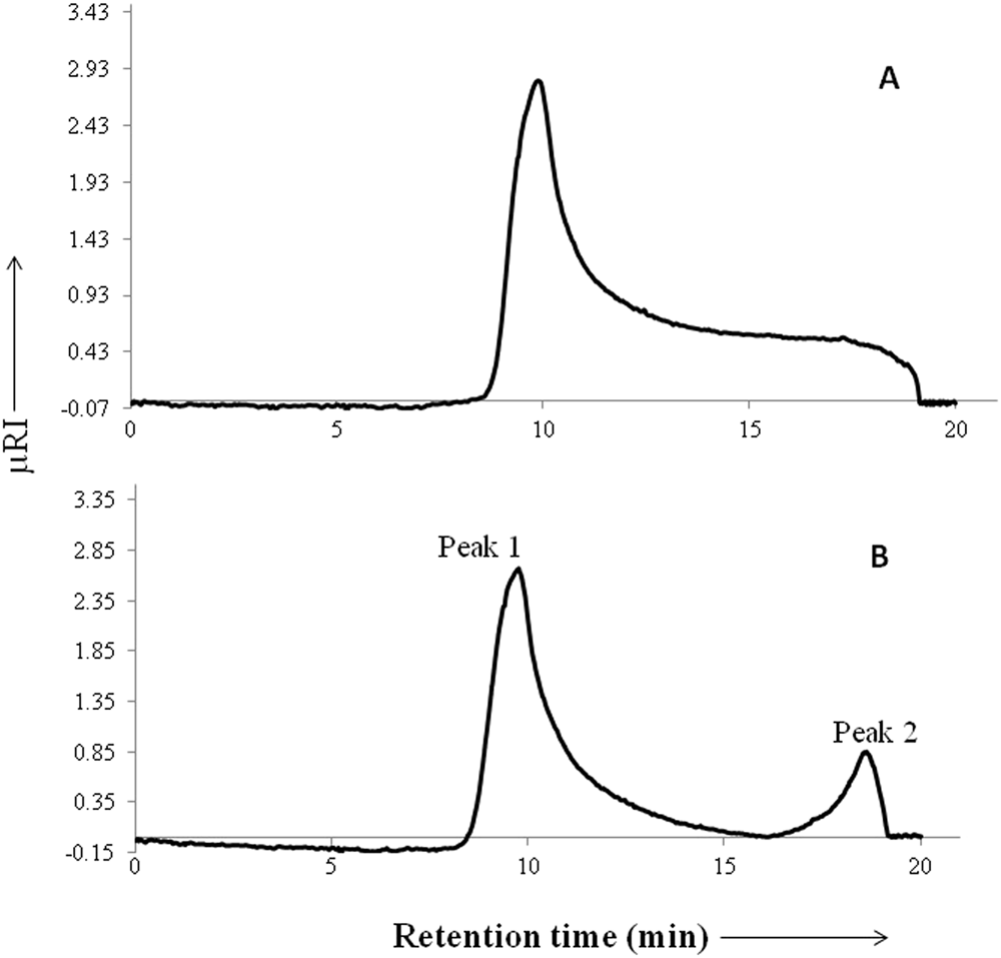
GPC profiles of: (**A**) Purified guar gum (PGG) and (**B**) Methylated guar gum (MGG).

The GPC chromatogram of PGG and MGG are depicted in Fig. 2. Chromatogram of PGG was characterized by a single peak with *M*_*w*_ 1.7 × 10^6^ Da (Fig. 2A). Previous studies on guar gum have reported *M*_*w*_ to be 2.7 × 10^6^ Da^21^ and 4 × 10^6^ Da^4^. The results obtained in the present study are thus in accordance with the already published data. The chromatogram of MGG, on the other hand, exhibited two peaks (peak 1 and 2) (Fig. 2B) having relative abundance of 86.20% and 13.80%, respectively. The *M*_*w*_ of peak-1 and peak-2 was found to be 1.9 × 10^6^ and 2.6 × 10^4^ Da, respectively. Peak-1 had *M*_*w*_ comparable to that of PGG. Appearance of relatively small amount (relative abundance) of low *M*_*w*_ peak (peak-2) could be attributed to depolymerization of polymer in basic conditions employed during methylation reaction. Poly dispersity index (PDI), which provides a measure of molecular weight distribution of polymer was determined for PGG as well as MGG. PDI for PGG was 1.15 while that for MGG was 1.32. A higher value of PDI for MGG could possibly be explained by base-induced degradation of PGG polymer.

### Small angle neutron scattering (SANS)

**Figure 3:**
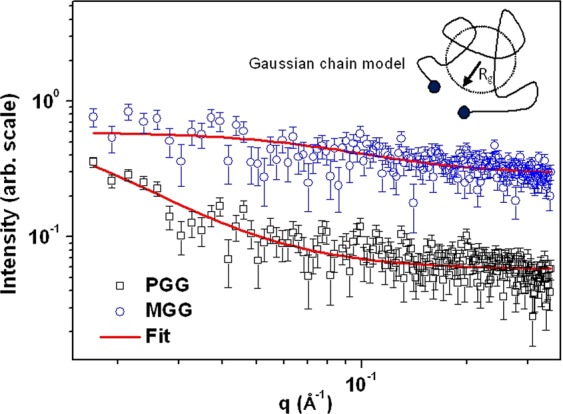
SANS profiles of the PGG and MGG. The solid line represents model fit to the data. Inset shows the Gaussian chain polymer model used for fitting the data.

Figure 3 shows the SANS profiles of the PGG and MGG dispersions, depicting dependence of the scattering intensity (I) on the scattering wave vector (q).

SANS provides structural information in mesoscopic length scale of 1–100 nm and is an excellent tool to probe the conformation and chain length of polymers in dispersion form, which is not possible with microscopy techniques. Scattering experiments provides structural information in reciprocal space and therefore it is an indirect technique to probe the structure. Angular distribution of scattered neutrons in narrow angle (<10°) bears information of the nano-scale objects. The dimensions in reciprocal and real space are inversely proportional, i.e. d~2π/q where d is the real space dimension and q is the reciprocal space dimension. This clearly indicates that larger objects scatter neutrons at lower q values whereas the smaller objects scatter neutrons at higher q values. Guar gum is reported to exhibit conformation similar to that of Gaussian chain^22^. The scattering data was therefore interpreted using Gaussian conformation of the polymer chain. Moreover, in order to get the quantitative information, scattering intensity for Gaussian polymer chain can be written as Eq. 7^23^.

7 $$I(q)=\frac{2{I}_{0}}{{x}^{2}}(x-1+{e}^{-x})$$

where x = R_g_^2^q^2^, I_0_ is q-independent scale parameter. R_g_ is the radius of gyration and is defined as the root mean square distance of the collection of atoms from their common center of gravity.

As evident from Fig. 3, the scattering profiles for PGG and MGG are significantly different. The scattering intensity at low q in PGG shows increasing trend whereas it remains constant for MGG. It indicates that chain length of polymer in PGG is significantly larger compared to that in MGG. The inset of Fig. 3 shows the Gaussian chain polymer model. Radius of gyration depends on the chain length of the polymer for a given conformation. The fitting of the scattering intensity data in Gaussian chain model (Eq. 7) is shown in Fig. 3. The estimated R_g_ values from the fitting of SANS profiles for PGG and MGG is found to be 102 (**±**22) Å and 19 (**±**3) Å, respectively. However, no change in conformation of the polymer was observed as a result of methylation. The SANS results clearly indicate that methylation of the GG causes reduction in the chain length thus substantiating viscosity and GPC data.

### Thermal behavior of PGG and MGG

**Figure 4:**
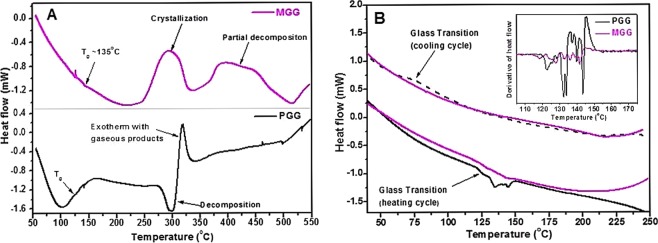
(**A**) DSC thermograms of PGG and MGG. (**B**) DSC thermograms of pre-heated PGG and MGG powders recorded during heating as well as cooling cycles. Inset shows the derivative plot of heat flow curve during heating cycle.

Figure 4 shows the DSC thermograms recorded on PGG and MGG powders over 35–550 °C. The curves are vertically spaced for clarity. It can be seen that PGG undergoes moisture loss during initial heating (up to 150 °C), followed by direct decomposition (T_onset_ ~ 280 °C, T_peak_ ~ 300 °C). The decomposition endotherm is followed by an overlapping exotherm (peaked ~ 319 °C), which may be attributed to enthalpy release during formation of gaseous decomposition products. The results obtained in the present study are in agreement with previous reports on guar gum wherein endothermic and exothermic peaks were observed at 296 °C and 317 °C, respectively^24^. Weight measurement of DSC residue confirms PGG decomposition with 80% weight lost during heating (Table 1). On the other hand, MGG powder does not show notable moisture loss during initial heating, indicating its relative stabilization towards hygroscopic/hydroscopic behavior upon methylation. This was further noted while storing the pre-heated PGG and MGG powders (heated up to 250 °C) in desiccators for 7 days. These powders, when subjected to DSC measurements again showed moisture loss for PGG but not for MGG.

**Table 1:** DSC measurements details on PGG and MGG samples and residue.

Sample	Sample Pan weight (mg)	Reference Pan weight (mg)	Sample Weight (mg)	Temperature Program and atmosphere	DSC Residue weight (mg) *(weight loss)*
PGG	48.48	48.41	1.43	50 °C to 550 °C @ 10 °C/min. Flowing Ar (60 ml/min.)	0.30 (*~80%*)
MGG	48.44	48.44	2.23	50 °C to 550 °C @ 10 °C/min. Flowing Ar (60 ml/min.)	0.98 (*~56%*)

Further observation of Fig. 4A indicates that unlike PGG, MGG does not undergo decomposition starting around 280 °C. Instead, it shows a broad exotherm (T_onset_ ~ 248 °C, T_peak_ ~ 294 °C). Such exotherms typically characterize crystallization of disordered amorphous materials. To confirm if the exotherm is due to decomposition or crystallization, MGG sample was kept at isothermal hold at 250 °C for 1 h in a furnace, followed by weight measurement. Results indicated no weight loss, thereby indicating that the exotherm centered at 294 °C is due to partial crystallization of MGG. These results indicate that upon methylation, guar gum is structurally modified to enhance thermal stability towards decomposition. It also indicates that the MGG structure is modified with relative higher degree of local ordering (as compared to PGG), which promotes crystallization upon heating. An increase in crystallinity after acrylation of GG was also reported earlier^12^. GG contains huge number of hydroxyl groups causing large number of random hydrogen bonds being formed. The increase in crystallinity could be due to reduction in such random hydrogen bonding, allowing increased ordering of particles. Further heating of MGG powder in DSC gives a second broad exotherm, easily identifiable in terms of two overlapping peaks (centered around 398 °C and 440 °C). Weight analysis of residue (Table 1) indicates ~56% loss, clearly indicating that decomposition of MGG occurs during this temperature range. Relatively higher fraction of DSC residue (~44% for MGG, ~20% for PGG) further indicates higher thermal stability of MGG compared to PGG. However, absence of any endothermic onset of decomposition in case of MGG (as seen for PGG) suggests vigorous decomposition with release of gaseous products having higher enthalpy of formation. As a result, exotherm associated with this enthalpy of formation, apparently masks the observation of endotherm associated with energy required for decomposition.

Since PGG primarily consist of amorphous matrix, we tried to analyze its DSC thermograms for identifying glass transition temperature and effect of methylation. Fig. 4B presents the heating and cooling cycle thermograms recorded on PGG and MGG over a limited temperature range, well below the onset of decomposition (35 °C to 250 °C). Curves are shown for samples pre-heated up to 250 °C in DSC instrument itself (under argon flow), so as to avoid the interference from moisture loss endotherm (100–150 °C). It is visible from the curves that both materials show glass transition behavior around 120 °C. A clear step-like glass transition trance is not observed for either of the studied materials, possibly due to loss of residual moisture around the same temperature range. However, attribution of weak endotherm around 120 °C to glass transition phenomenon (heating cycle) is supported by cooling cycle DSC trace, which show glass transition like signature for PGG, though at low temperature (~70 °C), due to thermal hysteresis. Both, PGG and MGG show glass transition around same temperature and no identifiable effect of methylation of guar gum could be noted. Glass transition signature could not be observed for MGG in cooling cycle, possibly due to relatively higher degree of structural order and also partial crystallization.

In summary, DSC studies confirm that PGG matrix is thermally stabilized upon methylation. While no clear shift in glass transition was observed, tendency of higher structural order and partial crystallization prior to decomposition was seen in methylated guar gum. It would be interesting to extend detailed thermal analysis of these materials so as to characterize their potential as packaging material.

### Effect of methylation on mechanical and barrier properties of films

Effect of addition of MGG in PGG on tensile strength of films is depicted in Table 2. A significant (p < 0.05) reduction in tensile strength of films was observed due to inclusion of MGG. Control PGG films had a tensile strength of 18.01 ± 1.6 MPa which reduced to 5.5 ± 1.5 MPa for films prepared with 100% MGG (Table 2). Young’s modulus defines the rigidity of films with higher values indicating higher rigidity of the films. The value of Young’s modulus for PGG films was 46.69 ± 2.65 MPa. As evident from Table 2, Young’s modulus of elasticity remained unaffected up to 25% replacement with MGG. The values were significantly reduced with increasing concentration of MGG. The values of Young’s modulus were 37.91 ± 5.17, 34.93 ± 3.19, 24.96 ± 2.01 MPa for 50, 75 and 100% MGG films, respectively.

**Table 2:** Mechanical properties of films prepared from various combinations of PGG and MGG.

Film	Tensile strength (MPa)	Young’s modulus (GPa)	Puncture strength (N)	% Elongation	WVTR (g/m^2^/d)	Opacity (%)
PGG	18.01 ± 1.64^a^	46.69 ± 2.65^a^	1.44 ± 0.21^a^	31.58 ± 0.84^d^	171.05 ± 5.55^ab^	13.44 ± 0.29^a^
10% MGG	11.31 ± 1.71^b^	47.39 ± 5.12^a^	1.38 ± 0.21^ab^	35.97 ± 0.45^c^	173.24 ± 0.92^a^	13.56 ± 0.08^a^
25% MGG	10.97 ± 0.16^b^	47.62 ± 4.63^a^	1.11 ± 0.12^bc^	37.01 ± 1.62^c^	157.36 ± 5.59^b^	13.48 ± 0.21^a^
50% MGG	10.46 ± 0.35^b^	37.91 ± 5.17^b^	1.00 ± 0.14^c^	47.06 ± 2.32^a^	157.36 ± 6.84^b^	13.60 ± 0.24^a^
75% MGG	7.47 ± 0.29^c^	34.93 ± 3.19^b^	1.28 ± 0.06^abc^	49.43 ± 0.93^a^	141.82 ± 3.73^c^	13.56 ± 0.23^a^
100% MGG	5.58 ± 0.46^c^	24.96 ± 2.01^c^	1.14 ± 0.09^bc^	44.89 ± 0.46^b^	102.05 ± 0.04^d^	13.00 ± 0.16^b^

Values in the same column followed by same superscript are not significantly (p < 0.05) different.

In several previous reports it has been demonstrated that for amorphous polymers, mechanical properties are directly proportional to the molecular weight of the polymer^25^. It has been postulated that higher molecular weight leads to higher number of chain entanglements per molecule^26^. Higher number of entanglements require more energy to break the entangled chains thereby increasing mechanical strength. Decreased mechanical properties of the films observed is due to reduction of molecular weight of the polymer during methylation as confirmed by GPC and rheological analysis. Previous work also demonstrated decreased mechanical properties of films prepared using irradiated GG due to reduction in molecular weight^4^. Apart from reduction in molecular weight, decrease in mechanical properties might be due to the reduction in intermolecular hydrogen bonding as evident from IR spectra. With respect to puncture strength, no trend could be observed due to addition of MGG (Table 2).

A significant (p < 0.05) improvement in percent elongation was observed with increasing concentration (up to 75%) of MGG (Table 2). Control PGG films had percent elongation of 31.5 ± 0.8%, which increased to 49.4 ± 0.9% at 75% MGG concentration. An improved percent elongation at break has also been reported earlier in composite films made up of starch in combination of various chemically modified derivatives such as hydroxypropyl and carboxymethyl cellulose, which was attributed to association of cellulose with swollen starch or leached amylose chains^27^. Interestingly, films prepared with 100% MGG although having percent elongation significantly (p < 0.05) higher than control PGG films, demonstrated significantly lower percent elongation as compared to films having 75% MGG (Table 2). This might be due to the fact that in present study glycerol is employed as a plasticizer. Since glycerol is polar in nature it might have low compatibility with non-polar MGG. Using non-polar plasticizers for film preparation with 100% MGG might result in better % elongation for these films.

Addition of MGG also had a significant (p < 0.05) effect on WVTR of films (Table 2). Control PGG films had a WVTR of 171 ± 15 g/m^2^/d. A 17% and 40% reduction in WVTR was observed in comparison to control for films prepared with 75% and 100% MGG, respectively. Observed reduction in WVTR could be due to introduction of hydrophobic methyl groups onto polymer backbone resulting in lesser water affinity. Similar results of decreased WVTR as a result of introduction of hydrophobicity in acetylated corn starch films has been reported earlier by various researchers^28–31^. The results presented here are thus in accordance with the literature data.

Opacity is an important attribute of packaging films as it affects visibility of the packaged products to consumers. It represents the degree to which light is not allowed to pass through the films. As shown in Table 2, opacity was unaffected up to 75% replacement with MGG but decreased in films containing 100% MGG. Thus the films made from 100% MGG were clearer and more transparent than films prepared from other compositions.

## Conclusions

A protocol for green synthesis of methylated guar gum was established that resulted in a degree of substitution as 0.4. A decrease in M_w_ and radius of gyration of guar gum was observed due to methylation. As evident from DSC experiments, MGG demonstrated better thermal stability, hydrophobicity and increased crystallinity as compared to PGG. MGG solutions demonstrated lower viscosity and CI as compared to PGG. However, due to lower M_w_, MGG containing films had lower tensile strength as compared to PGG. Films prepared with partial replacement of PGG with MGG demonstrated increased barrier to water vapor transmission due to hydrophobic nature of MGG. Results obtained in present study indicate that chemical modifications such as methylation can be helpful in improving thermal stability and barrier properties.

## Supplementary information

Supplementary tables
