# Supplementary material for: Methylation of guar gum for improving mechanical and barrier properties of biodegradable packaging films
Source: Sci Rep. 2019 Oct 10;9:14505. doi: 10.1038/s41598-019-50991-7 (PMC6787019; doi:10.1038/s41598-019-50991-7)
Supplement: Supplementary file 1 — Supplementary tables [file 41598_2019_50991_MOESM1_ESM.docx]

**Methylation of guar gum for improving mechanical and barrier properties of biodegradable packaging films**

**Jyoti Tripathi*, Rupali Ambolikar, Sumit Gupta*, Dheeraj Jain, Jitendra Bahadur and Prasad Shekhar Variyar.**

Table S1. Formulation reference for methylation of guar gum (DS=0.4)

| Component | Weight (g) | No. of moles (n) | Molar ratio component w.r.t. saccaride unit |
| --- | --- | --- | --- |
| Guar gum | 2.5 | 0.017 | 1 |
| NaOH | 6.25 | 0.156 | 9.17 |
| Methyl iodide | 4.54 | 0.032 | 1.88 |

Table S2. Elution time (min) of pullulan standards in GPC

| S.No. | Molecular weight of pullulan | Retention time (min) |
| --- | --- | --- |
| 1 | 6 K | 19.005 |
| 2 | 10 K | 18.84 |
| 3 | 21.7 K | 18.33 |
| 4 | 48.8 K | 17.475 |
| 5 | 113 K | 16.06 |
| 6 | 210 K | 14.535 |
| 7 | 366 K | 12.765 |
| 8 | 805 K | 11.075 |
| 9 | 1600 K | 10.135 |
| 10 | 2560 K | 9.655 |
